# Triage knowledge and skills among nurses in emergency units of Specialized Hospital in Hawassa, Ethiopia: cross sectional study

**DOI:** 10.1186/s13104-019-4062-1

**Published:** 2019-01-14

**Authors:** Bereket Duko, Ephrem Geja, Zewdie Oltaye, Fanuel Belayneh, Addisu Kedir, Melese Gebire

**Affiliations:** 1 Faculty of Health Sciences, College of Medicine and Health Sciences, Hawassa University, P.O. Box 1560, Hawassa, Ethiopia; 2 Hawassa University Comprehensive Specialized Hospital, P.O. Box 1560, Hawassa, Ethiopia

**Keywords:** Triage, Triage skills, Triage knowledge, Nurses, Ethiopia

## Abstract

**Objective:**

This study was aimed to assess knowledge and skills of triage and associated factors among nurses in emergency department of Hawassa University Comprehensive Specialized Hospital, South Ethiopia. Institutional based cross-sectional study design was conducted among 101 nurses from March 1–30, 2018. The data was coded and entered to SPSS version 22.0. Descriptive statistics was done and Chi square test was done to show the association between independent variables and dependent variable.

**Results:**

Among the study participants, 57.4% were female and 87% were in age group of ≤ 30 years. 51.5% had low triage knowledge scores, with the mean score being 9.54 (SD = 2.317), 76.2% perceived their overall triage skill to be at good level, with mean score 95.75 (SD = 9.562). Working experience of study participants (χ^2^ = 15.204, p < .01), Educational level of study participant (χ^2^ = 22.148, p < .01) and triage experience (χ^2^ = 13.638, p < .01) were factors associated with triage knowledge. Working experience (χ^2^ = 7.944, p < .05) and triage experience (χ^2^ = 6.264, p < .05) were factors associated with triage skill.

**Electronic supplementary material:**

The online version of this article (10.1186/s13104-019-4062-1) contains supplementary material, which is available to authorized users.

## Introduction

Emergency department (ED) generally provides immediate care for 24 h every day. The erratic numbers of patients coming to ED suffer from various conditions with unknown severity, urgency, and definite diagnosis [1]. The patients who are suffering from life threatening conditions, such as cardiac arrest, airway obstruction, and shock should be prioritized to provide them an early immediate care to save their lives. Nerveless, the crowding of patients visiting to ED can have an impact on the quality of care by diversifying the resources intended for patients, which are in need of emergency care compared to the individuals who have potentially less urgent needs [1, 2].

Triage is putting the patient in the right place at the right time to receive the right level of care and the allocation of appropriate resources to meet the patient’s medical needs’. This place of the hospital allows for assignment of the care taker to suitable assessment and treatment place [3–9].

A study from South Africa showed that the objective measure of knowledge improved significantly after the training workshop given to them [10]. Another study from Tanzania across revealed that 52% and 58% of the respondents involved in the study failed to allocate proper patient’s triage category and had no knowledge on waiting time limits for patients’ triaged categories [3].

Ethiopia also lacks human resource with the knowledge and essential skills to support a coordinated emergency medical care system. Moreover, this situation together with the lack of facilities, equipment and some basic infrastructure for delivering emergency care makes difficult to provide dedicated emergency care with appropriate triage protocols, rapid diagnosis and timely treatment [5].

Therefore, it is necessary to examine emergency nurses’ triage knowledge and skills including other related factors. Thus, this study was aimed at assess knowledge and skills of triage and associated factors among nurses working in emergency departments of Hawassa University Comprehensive Specialized Hospital, Hawassa, Ethiopia.

## Main text

### Study setting and population

Institutional based cross-sectional study design was conducted in Hawassa City, which is located 273 km far from capital city of Ethiopia, Addis Ababa. Hawassa University Comprehensive Specialized Hospital staff nurses’ were included in the study. Single population proportion formula was used to calculate sample size (101 nurses). A systematic random sampling technique was used to recruit study participants.

### Data collection instrument

The structured questionnaire was constructed by researchers which included socio-demographic related questions, was based on existing literature on triage in emergency care (EC) and the advice of experts in emergency care who had been consulted. The data collection tool had the following parts, a structured questionnaire on socio-demographic and clinical experiences, knowledge assessment questions and a triage skill assessment questionnaire. The socio-demographic and knowledge assessment questionnaire consisted of 17 questions, covering the following; demographic data & triage knowledge questions. We had conducted a pretest to check the knowledge questionnaire for internal consistency and reliability and obtained Cronbach's alpha of .91. Triage skill was measured using triage skill questionnaire (TSQ). This questionnaire was which consists of 37 questions with three dimensions, including rapid assessment, patient categorization, and patient allocation. The study participants were asked to respond to each item using 1–5 rating scale: 1 = need improvement, 2 = poor, 3 = fair, 4 = good, and 5 = very good and its total score summation ranges from 37–185; the final result was converted to a percentage. The final score was interpreted as low level triage skill (scored—60%), moderate level score (scored—60–80%), high level score (scored > 80%). The triage skill questionnaire was highly reliable in the study with Cronbach’s alpha coefficient of .95 [11] (see also additional file 1).

### Data processing and analysis

Data was compiled, entered and analyzed using SPSS version 22. Percentages and frequency was calculated and p-value with 95% confidence interval was calculated to assess association between dependent and independent variables. A p-value of less than .05 was considered statistically significant, and adjusted odds ratio with 95% CI and Chi-square was calculated to determine association. Finally, data was presented by using numbers, frequencies, tables, charts and figures.

### Result

#### Socio-demographic characteristics of the respondents

A total 101 study participants were included in the study. The mean age of study participants was 28.50 years (±SD = 4.32). Among the study participants; 57.4% were females, 86.1 were under 30 years, 67.3% were completed bachelor degree of nursing, 79.2% of the respondents with working experience of < 3 years, 19.8% had successfully completed the Triage Officer Course, 6.9% had attended the Basic Trauma Life Support (BTLS) training course and 7.9% had attended trauma in nursing care (Table 1).

**Table 1:** Training experience, work experience in Emergency department and triage experience of nurses in Three Hospitals, South Ethiopia, 2018

Variable	Category	Frequency	Percentage (%)
Age	Male	43	42.6
	Female	58	57.4
Sex (years)	≤ 30	87	86.1
	> 30	14	13.9
Education	Diploma in nursing	26	25.70
	BSc. in nursing	88	67.30
	MSc in nursing	7	6.90
Training experience	Basic Trauma Life Support (BTLS)	7	6.9
	Basic Cardiac Life Support (BCLS)	6	5.9
	Triage Officer Course (ToC)	20	19.8
	Trauma in Nursing Care (TNC)	8	7.9
	Emergency Care	16	15.8
	Disaster Management	6	5.9
	Others	4	3.96
Work experience in emergency department (years)	< 3	80	79.2
	3–5	15	14.9
	≥ 5	6	5.9
Triage experience (years)	≤ 3	98	97
	≥ 3	3	3

#### Triage knowledge and skill

**Fig. 1:**
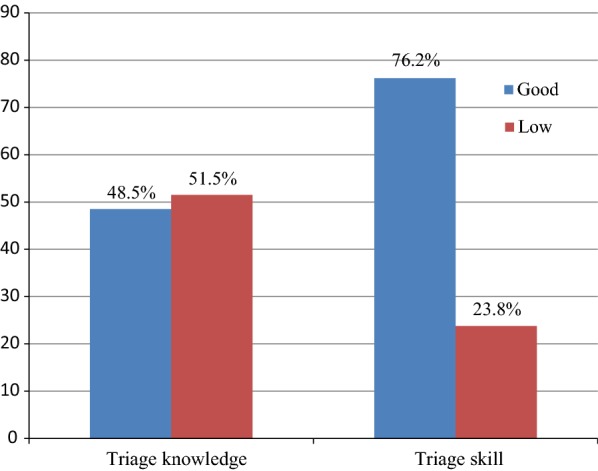
Distribution of triage knowledge and triage skill among nurses working in emergency department, Hawassa, 2018

51.5% and 76.2% of the respondents had low triage knowledge scores, with the mean score being 9.54 (SD = 2.32) and perceived their overall triage skill to be good, the mean score of 95.75 (SD = 9.56) respectively (Fig. 1).

#### Factors associated with triage knowledge and skill

Logistic regression analysis revealed working experience of study participants (χ^2^ = 15.204, p < .01), Educational level of study participant (χ^2^ = 22.148, p < .01) and triage experience (χ^2^ = 13.638, p < .01) were factors significantly associated with triage knowledge. While, working experience (χ^2^ = 7.944, p < .05) and triage experience (χ^2^ = 6.264, p < .05) were factors significantly associated with triage skill (Table 2).

**Table 2:** Associated factor of triage knowledge and skill among nurses working in emergency department of the hospitals, Ethiopia, 2018 G.C

Variable	Test	Association					
		Age	Gender	Working experience	Educational level	Training experience	Triage experience
Triage knowledge	Chi-square	.208	1.327	15.204	22.148	.892	13.638
	P-value	.648	.249	.002***	.000***	.345	.000***
Triage skill	Chi-square	3.271	1.100	7.944	.917	.095	6.264
	P-value	.071	.294	.047**	.632	.758	.012**

** Significant at .05 (2-sided)

*** Significant at .01 (2-sided)

### Discussion

This study revealed that 48.5% of triage nurses were found with poor knowledge of triage and 23.8% of study participants perceived they as inadequately prepared for triage skill. This result is comparable to the study conducted Tanzania, Indonesia, Gutamala, South Africa and other countries [3, 10–14]. The reason might be due to the majority nurses who are working in emergency department didn’t attend training course special to triage knowledge and work experience of nurses might contribute to their knowledge. Nevertheless, the current finding is lower than the studies done in Sweden, Indonesia and Switzerland 59.6% [3, 4, 14, 15]. The variation might be due to difference in sample size (large sample size versus small sample size), data collection tool meaning some studies used Canadian Triage and Acuity scale, Emergency Triage Assessment and Treatment (ETAT) guidelines, Specific and Timely Appointments for Triage (STAT) and South African triage assessment scales while our study used a structured knowledge assessment tool and a triage skill questionnaire (TSQ).

Work experience of nurses is a factor which contributes to triage knowledge. In this study, 79.2% of the respondents had less than 3 years work experience in emergency department. The finding is in agreement with finding from Pakistan study. However, the finding is lower than a study conduct in [10, 16]. This variation might be due to regular duty rotation is practiced in the study setting which made nurses not to stay in emergency department for a longer period.

Having training on triage has significant association with triage skill. This is evidenced by having training might be skilled them to practice properly. Triage knowledge and skill of the study participant was affected by work experience of nurses, educational status, having triage training and triage experience. The finding is in congruent with other studies [3, 16–18]. This also supported by the fact that being experienced, increased educational status, having triage training and triage experience might capacitate the skill capture among nurses.

### Conclusion

The triage skill was found to be at good level while the triage knowledge was at a low level. In addition, there were associations between triage skill, working experience and triage experience. There were also association between triage knowledge, working experience, Educational level and triage experience. Ministry of health and hospitals should provide trainings and education to strengthen triage knowledge and skill.

## Limitation of the study

The study used small sample size for our study, this might over or under estimate the knowledge and skill of the nurses.

## Additional file

**Additional file 1.** Questionnaire to assess knowledge and skills of triage among nurses workingin the emergency units, specialized Hospital in Hawassa, Ethiopia.

## References

[1] Considine J, Botti M, Thomas S (2007). Do knowledge and experience have specific roles in triage decision-making?. Acad Emerg Med.

[2] Iserson K, Moskop J (2007). Triage in medicine, part I: concept, history, and types. Ann Emerg Med.

[3] Kelly AM, Richardson D (2001). Training for the role of triage in Australasia. Emerg Med..

[4] Mallett J, Woolwich C (1990). Triage in accident and emergency departments. Adv Nurs.

[5] Pozner CN, Bayleygne TM, Davis MA (2003). Emergency medical services capacities in the developing world: preliminary evaluation and training in Addis Ababa. Ethiopia. Prehosp Emerg Care..

[6] Considine J, LeVasseur SA, Villanueva E (2004). The Australasian Triage Scale: examining emergency department nurses’ performance using computer and paper scenarios. Ann Emerg Med..

[7] ElGammal ME (2014). Emergency department triage. Why and how?. Saudi Med J.

[8] Gerdtz M, Bucknall T (2000). Australian triage nurses’ decision-making and scope of practice. Aust J Adv Nurs..

[9] Qureshi NA (2010). Triage systems: a review of literature with reference of Saudi Arabia East Mediterr Health J..

[10] Naidoo M (2017). An evaluation of the emergency care training workshops in the province of KwaZulu-Natal, South Africa. Afr J Prim Health Care Fam Med..

[11] Fathoni M, Sangchan H, Songwathana P (2010). Triage knowledge and skills among emergency nurses in East Java Province, Indonesia. Aust Emerg Nurs J..

[12] Kapoor R, Sandoval MA, Avendaño L, Cruz AT, Soto MA, Camp EA (2016). Regional scale-up of an Emergency Triage Assessment and Treatment (ETAT) training programme from a referral hospital to primary care health centers in Guatemala. BMJ Emerg Med J.

[13] Göransson KE, Ehrenberg A, Marklund B, Ehnfors M (2006). Emergency department triage: is there a link between nurses’ personal characteristics and accuracy in triage decisions?. Accid Emerg Nurs..

[14] Jordi K, Grossmann F, Gaddis GM, Cignacco E, Denhaerynck K, Schwendimann R, Nickel CH (2015). Nurses’ accuracy and self-perceived ability using the Emergency Severity Index triage tool: a cross-sectional study in four Swiss hospitals. Scand J Trauma Resusc Emerg Med..

[15] Purnell L (1993). A survey of qualifications, special training, and levels of personnel working in emergency department triage. J Nurs staff develop..

[16] Fathoni M, Sangchan H, Songwathana P (2013). Relationships between triage knowledge, training, working experiences and triage skills among emergency nurses in East Java, Indonesia. Nurse Media J Nurs.

[17] Afaya A, Azongo TB, Yakong VN (2017). Perceptions and knowledge on triage of nurses working in emergency Departments of Hospitals in the Tamale Metropolis Ghana. IOSR JNHS..

[18] Carpenter CR, Bromley M, Caterino JM, Chun A, Gerson LW, Greenspan J, Hwang U, John DP, Lyons WL, Platts-Mills TF (2014). Optimal older adult emergency care: introducing multidisciplinary geriatric emergency department guidelines from the American College of Emergency Physicians, American Geriatrics Society, Emergency Nurses Association, and Society for Academic Emergency Medicine. J Am Geriatr Soc..

