# Supplementary material for: Triage knowledge and skills among nurses in emergency units of Specialized Hospital in Hawassa, Ethiopia: cross sectional study
Source: BMC Res Notes. 2019 Jan 14;12:21. doi: 10.1186/s13104-019-4062-1 (PMC6332676; doi:10.1186/s13104-019-4062-1)
Supplement: Supplementary file 1 — Additional file 1. Questionnaire to assess knowledge and skills of triage among nurses workingin the emergency units, specialized Hospital in Hawassa, Ethiopia. [file 13104_2019_4062_MOESM1_ESM.docx]

**ADDITIONAL FILE 1**

**DATA COLLECTION TOOL**

QUESTIONNAIRE TO ASSESS KNOWLEDGE AND SKIILS OF TRIAGE AMONG NURSES WORKING IN THE EMERGENCY UNITS, SPECIALIZED HOSPITAL IN HAWASSA

**Socio-demographic Data**

1. **Demographic Data Sheet (DDS) Code: … … … … … … … …**

**Instruction**

I would like to ask you some information regarding personal data. Please answer by putting marking ( x) in the space available as that is appropriate for you and/or filling in the blank.

1. Age … … … … years

2. Gender: 1 Male 2 Female

3. Educational level;

1 Diploma in Nursing 2 Bachelors in Nursing

3 Postgraduate in Nursing 4 Others specify...............................

4. Working experience by nursing Profession………………years…………months

5. How long have you been working as a nurse in ED? … … .Years… … months

6. How long have you been working as a nurse in triage room…….years… … months

7. Do you currently work in triage room? Yes no

8. Training and continuing education

8.1 Did you attend any training, course or workshop during the past three years?

Yes no

Table 1; Types of training with its number and duration in days.

| **No** | **Training** | **Number**  **of**  **training** | **Duration**  **of training**  **course(days)** |  | |
| --- | --- | --- | --- | --- | --- |
|  |  |  |  |  |  |
| 1 | Basic Cardiac Life Support(BCLS) |  |  |  |  |
| 2 | Basic Trauma Life Support (BTLS) |  |  |  |  |
| 3 | Triage Course |  |  |  |  |
| 4 | Trauma in Nursing Care |  |  |  |  |
| 5 | Emergency Care |  |  |  |  |
| 6 | Disaster Management |  |  |  |  |
| 7 | Other…… … … …(Please identify it) |  |  |  |  |

**Knowledge of triage: this section assessed the participants’ knowledge of triage.**

1. Define triage ...................................................................................................................................................................................................................................................................................
2. How long should a patient with the following color code wait for treatment in the casualty or emergency unit.(select your answer from here: less than 10min, immediate, less than 60 min, less than 240min)
   1. Red………………………………..
   2. Orange…………………………….
   3. Yellow……………………………..
   4. Green……………………………..

**Assume you are at the casualty or emergency unit as a triage nurse. Assign priorities to the following patients who come for emergency health care services with the following conditions. (*Label as P1, P2, P3: where P1 stands for first priority, P2 second priority, and P3 third priority*)*.***

1. An Adult patient. Breathing with difficult. Respirations of 8/min, Systolic blood pressure of 80mmHg……………….
2. An Adult patient. Fast breathing. Coughing. Respirations of 40/min, Temperature of 39 degrees centigrade …………………
3. An Adult patient. Face burn. On severe pain. Respirations of 29, Pulse rate of 129/min…………
4. Female. Pregnancy. walking. Trauma. Respirations of 20/min, Pulse rate of 100/min, and Systolic blood pressure of 130mmHg………………..
5. Adult patient. Walking. Feeling discomfort. Headache. Systolic blood pressure 200mmHg………………….
6. Adult patient. Skin moderate pale, cool, and dry. Reacts to voice. Respirations of 28/min, pulse rate of 110/min, Systolic blood pressure of 90mmHg………………..
7. Adult patient on stretcher. Controlled bleeding. Weak peripheral pulse. Respirations of 20/min, pulse rate of 100/min, Systolic blood pressure of 130mmHg………………….
8. Adult patient with chest pain radiating to the left arm. Walking. Respirations of 30/min, pulse rate of 110/min, Systolic blood pressure of 90mmHg……………

**Triage Skill Questionnaire (TSQ)**

Instruction: Please assess your own ability in triage skills by check list the number on scale 1 to 5 following each statement below. There are five options available: **5 = very good, 4 = good, 3 = fair, 2 = poor, 1 = need, improvement**

| **No** | **Triage skills** | **Perceived Triage Skill** | | | | |
| --- | --- | --- | --- | --- | --- | --- |
|  |  | Very good  (5) | Good  (4) | Fair  (3) | Poor  (2) | Need improvement  (1) |
|  | **Rapid Patient Assessment** |  |  |  |  |  |
| **1** | Aassess patient include vital signs with rapid assessment in 2-5 minutes |  |  |  |  |  |
| **2** | Aassess or ask chief complaint of the patient rapidly |  |  |  |  |  |
| **3** | In unconscious patients, look in the upper airway such as blood, vomit, foreign bodies, oedema, and tongue obstruction as assess airway patency |  |  |  |  |  |
| **4** | Decide to open airway and remove foreign body when airway is obstructed according to airway management (A) |  |  |  |  |  |
| **5** | Give positioning airway to maintain patency by chin lift |  |  |  |  |  |
| **6** | Perform clear airway by correct position with jaw trust and head tilt |  |  |  |  |  |
| **7** | Perform clear airway by correct position by jaw trust without head tilt if the patient suspect cervical spinal |  |  |  |  |  |
| **8** | Perform to insert oropharyngeal or nasopharyngeal airway |  |  |  |  |  |
| **9** | Look at the chest about patient chest abnormal movement |  |  |  |  |  |
| **10** | Assess rate and depth of respirations to observe (B) breathing rate and pattern rhythm with look and listen |  |  |  |  |  |
| 11 | Look at the patient skin to investigate for integrity, wounds, bruising, texture, and color |  |  |  |  |  |
| 12 | Listen the noise in the airway such as gurgling, snoring, and wheezing |  |  |  |  |  |
| 13 | Listen the silent or noisy breathing |  |  |  |  |  |
| 14 | Feel air blow from the patient with my cheek |  |  |  |  |  |
| 15 | Administer oxygen therapy |  |  |  |  |  |
| 16 | Perform manual ventilation |  |  |  |  |  |
| 17 | Perform bag-valve-mask ventilations |  |  |  |  |  |
| 18 | Protect cervical spine when patient suspect fracture cervical with cervical collar |  |  |  |  |  |
| 19 | Check pulse rate and rhythm according circulation assessment (C) |  |  |  |  |  |
| 20 | Assess of the capillary refill |  |  |  |  |  |
| 21 | Assess the temperature the patients |  |  |  |  |  |
| 22 | Assess the patient with diaphoresis |  |  |  |  |  |
| 23 | Perform chest compressions in critical condition of the patient |  |  |  |  |  |
| 24 | Collaborative with physician to administer emergency drugs |  |  |  |  |  |
| 25 | Assess internal and external bleeding |  |  |  |  |  |
| 26 | Perform control blood loss appropriately to stop bleeding the patient |  |  |  |  |  |
| 27 | Collaborate resuscitation to provide appropriate intravenous fluid |  |  |  |  |  |
|  | **Patient categorization** |  |  |  |  |  |
| 28 | Catagorization the patient according to triage priority |  |  |  |  |  |
| 29 | Identify the patient who require immediate care, urgent, and non urgent according to triage categorization |  |  |  |  |  |
| 30 | Avoid the condition of the patient with over-triage and under-triage |  |  |  |  |  |
| 31 | Initiation nursing intervention during triage categorization |  |  |  |  |  |
|  | **Patient allocation** |  |  |  |  |  |
| 32 | Make a decision to allocate the patient with priority 1 (Resuscitation in ED) in the right place |  |  |  |  |  |
| 33 | Make a decision to allocate the patient with priority 2  (Critical care in ED) in the right place |  |  |  |  |  |
| 34 | Allocate make a decision to allocate to the patient with priority 3 in the right place  (Ambulatory in ED) correctly |  |  |  |  |  |
| 35 | Allocate the patient with nursing intervention safety in ED |  |  |  |  |  |
| 36 | Allocate the patient by collaboration with other emergency nurse & medical doctor with hand over effectively |  |  |  |  |  |
| 37 | Allocate the patient to get advance treatment in ED in accurately and timely |  |  |  |  |  |
